# Adolescent idiopathic scoliosis and comorbid anxiety and depression: advances in psychophysiological interaction mechanisms and integrated interventions

**DOI:** 10.3389/fpsyt.2026.1878400

**Published:** 2026-10-28

**Authors:** Zhaomeng Hou, Jiaojiao Wu, Hua Lu, Chaoqun Chen, Jing Wang, Tingliang Han

**Affiliations:** 1 Department of Orthopedics and Traumatology, Yancheng TCM Hospital Affiliated to Nanjing University of Chinese Medicine, Yancheng, China; 2 Department of Orthopedics and Traumatology, Yancheng TCM Hospital, Yancheng, China; 3 Department of Pharmacology, School of Basic Medical Science, Nanjing Medical University, Nanjing, China

**Keywords:** adolescent idiopathic scoliosis, anxiety, comorbidity, depression, intervention, psychophysiological interaction

## Abstract

Adolescent Idiopathic Scoliosis (AIS) is a common three-dimensional spinal deformity, and its comorbidity with psychological disorders such as anxiety and depression is increasingly attracting clinical and research attention. This narrative review aims to summarize the epidemiological characteristics of this comorbidity in AIS. It explores the complex underlying psychophysiological interaction mechanisms, including psychosocial stress, chronic pain, body image disturbance, and interconnected neuroendocrine and neuroimmune pathways. Based on this mechanistic understanding, the article further synthesizes and evaluates existing intervention strategies for this comorbid condition, encompassing psychological therapies, pharmacotherapy, physical rehabilitation, and emerging multidisciplinary integrated care models. Finally, the review outlines future research directions, with the goal of providing a theoretical foundation and a comprehensive management framework to improve the holistic physical and mental health outcomes for individuals with AIS.

## 1 Introduction

Adolescent idiopathic scoliosis (AIS) is the most prevalent spinal deformity occurring during the critical developmental period of adolescence. The diagnosis and management of this condition, coupled with the visible postural changes, functional limitations, and long-term health concerns it entails, constitute a significant psychosocial stressor for affected individuals. A growing body of evidence underscores that AIS is not merely a physical deformity but is intricately linked with substantial psychological burden. To accurately interpret this evidence, it is essential to define the main outcomes examined in this review. These include: (1) formally diagnosed mental health disorders (e.g., clinical depression, anxiety disorders) based on clinical interviews or validated diagnostic criteria; (2) symptom severity, as measured by validated screening tools (e.g., patient-reported depression or anxiety scales) without implying a formal diagnosis; (3) positive screening scores, which indicate elevated risk or probable caseness but not confirmed psychiatric illness; (4) body-image perceptions and related distress, often assessed through disease-specific instruments; and (5) health-related quality of life (QoL) scores, such as those from the Scoliosis Research Society-22 (SRS-22), which include subscales for mental health and self-image but are not equivalent to psychiatric diagnoses.

Epidemiological studies consistently reveal elevated rates of co-occurring mental health disorders in this population. A national survey utilizing the ResearchMatch platform found that over half (58%) of adolescent scoliosis patients reported having received a mental health diagnosis. Among the 92 patients who had received a mental health diagnosis, clinical depression (83%), anxiety (71%), and negative body image (62%) were the most prevalent conditions (1). Notably, 76% of these diagnoses were made after the scoliosis diagnosis, suggesting a temporal link. The psychological comorbidity extends across gender and age groups, with network analysis identifying “excessive worry” and “sad mood” as central symptoms within the depression-anxiety symptom networks of AIS patients (2). The burden is further magnified in individuals with intersecting identities, such as those with concurrent gender dysphoria (GD), who exhibit substantially higher rates of psychological diagnoses—including a 7-fold increase in depression and a 12-fold increase in suicidal ideation—compared to those with AIS alone (3).

This comorbidity arises from a complex, dynamic interplay of psychological, social, neurobiological, and disease-specific factors. Chronic pain is a important factor in this psychophysiological interaction. In a cohort of Saudi adolescents with AIS, pain intensity was the most substantial contributor to reduced QoL, accounting for significant variability in SRS-22 scores (4). This pain often persists into adulthood; research on young adults with conservatively treated AIS showed that a significant proportion experienced back pain, with its development being strongly associated with psychological factors such as poor self-image and depression during adolescence (5). The relationship between pain and psychological state may be bidirectional. For instance, kinesiophobia (fear of movement due to pain) has been validated in AIS populations and shows significant correlations with both pain intensity and emotional conditions like depression, particularly in adult patients (6). Furthermore, psychological makeup itself may be linked to the future development of pain, hinting that adolescent mental health could serve as an early indicator for chronic pain in adulthood (5). Beyond pain, distorted self-image and concerns over physical appearance constitute another core disease-specific stressor. Research confirms that scoliosis severity, the patient’s perception of cosmetic deformity, and depression levels are independent predictors of health-related QoL (7). This is compounded by the psychosocial challenges of adolescence, where social acceptance and body image are paramount. Qualitative studies reveal that adolescents with AIS grapple with stressors related to disease- and treatment-induced changes, engage in various cognitive assessments of their condition, and employ diverse coping strategies, with their overall psychological well-being ranging from deterioration to resilience (8). The experience of bracing, in particular, introduces distinct stressors. Studies adapting stress questionnaires have identified brace-induced stress, which is often linked to anxious feelings about how one is perceived by others. This complex web of interactions suggests that psychological distress in AIS may be both a consequence and a contributor to the physical disease burden, potentially influencing key outcomes such as treatment adherence, rehabilitation efficacy, and long-term life satisfaction.

The notable prevalence and complex interplay of psychopathology in AIS suggest a move toward integrated management that concurrently addresses both spinal deformity and patients’ psychological well-being. The impact of untreated mental health conditions on clinical outcomes is substantial. In the surgical context, patients with preoperative mental health disorders (MHDs) experience higher postoperative pain, require more opioid analgesia during recovery, and have increased total healthcare utilization and costs, despite similar rates of surgical complications. Preoperative anxiety and depression have also been associated with increased hospital length of stay (9). These findings highlight that psychological factors are not peripheral concerns but are central to recovery trajectories. Similarly, in conservative management, psychological stress has been associated with treatment adherence and outcomes. Brace adherence, a critical determinant of preventing curve progression, is heavily influenced by psychosocial factors such as body image perception, stress, and family dynamics (10). Lower pre-bracing health-related QoL has been longitudinally associated with increased psychological stress during brace treatment (11). Fortunately, emerging evidence supports the potential efficacy of integrated interventions. Psychosocial interventions, including cognitive-behavioral therapy (CBT) and discourse-based psychological interventions, have shown feasibility and positive effects on postoperative pain, anxiety, coping abilities, and brace use. Technological innovations, such as mobile health applications for real-time monitoring of brace wear and patient-reported experiences (e.g., discomfort, emotional distress), offer promising tools for early detection of adherence barriers and timely clinical support. Furthermore, specific exercise therapies, like Schroth-based physiotherapy, have demonstrated benefits not only for correcting Cobb angles and trunk rotation but also for improving domains of the SRS-22, including self-image and mental health (12). These approaches underscore the importance of a holistic, patient-centered model. Effective management may therefore benefit from moving beyond a purely biomechanical focus to incorporate routine mental health screening, psychological support, and strategies that enhance resilience and social support, which have been associated with improved mental health outcomes (13). By adopting an integrative perspective, healthcare providers can optimize both the physical and psychological trajectories for adolescents navigating the challenges of idiopathic scoliosis.

Literature for this narrative review was identified through searches of PubMed, Web of Science, and Scopus using combinations of terms relating to AIS, anxiety, depression, pain, psychosocial functioning, and psychophysiological mechanisms. Searches included literature published up to April 2026. Priority was given to peer-reviewed studies directly involving patients with AIS, while relevant studies from other populations were included where they informed proposed mechanisms. As this was a narrative review, study selection was not intended to be exhaustive and no formal risk-of-bias assessment was conducted.

## 2 Epidemiology and clinical features of comorbidity of AIS with anxiety and depression

### 2.1 Comorbidity prevalence and associated factors

Cross-sectional and cohort studies consistently demonstrate that the prevalence of screen-identified anxiety and depressive symptoms is significantly higher among adolescents with AIS compared with healthy peers, with reported estimates typically ranging from 20% to 40% (14, 15). This range refers to elevated symptom scores on psychosocial screening tools rather than formally diagnosed psychiatric disorders. A national survey utilizing the ResearchMatch platform found that 58% of adolescent scoliosis patients had received a formal mental health diagnosis. Among those with a mental health diagnosis (92 out of 158 respondents), the most common diagnoses were clinical depression (83% of this subgroup), anxiety (71%), and negative body image (62%) (1). It is crucial to distinguish these diagnosed conditions from symptom severity or screening positivity. Network analysis of 765 AIS patients with elevated symptom scores identified “excessive worry” and “sad mood” as central and highly interconnected symptoms within the depression-anxiety network, underscoring their clinical significance (2). The burden is not transient; studies of adults with a history of AIS show that psychological distress, including poor self-image and depression during adolescence, are associated with the development of chronic back pain in young adulthood (5). Furthermore, the presence of a preoperative MHD is documented in approximately 16% of AIS patients undergoing surgery, a rate that remains stable in the postoperative year (16).

Several disease-specific and demographic factors are associated with a higher likelihood of this comorbidity. Greater scoliosis severity, often quantified by a larger Cobb angle, is a consistent correlate. Research indicates that Cobb angle, along with the degree of anterior trunk rotation and the patient’s perception of cosmetic deformity, are independent determinants of poorer health-related QoL, which is closely linked to psychological distress (7). In adults with unoperated AIS, a positive linear correlation exists between the Oswestry Disability Index (ODI) scores and both age and curve size (17). The treatment phase itself constitutes a critical risk period. Patients undergoing brace treatment or those awaiting surgery are particularly susceptible. A narrative review of the literature highlights the association of brace treatment with elevated anxiety, and concerns about appearance, discomfort, and social stigma can significantly hinder compliance and psychological well-being (14, 18). While one study comparing braced and observed patients found no significant between-group differences in psychosocial patient-reported outcomes over two years, it noted that depressive symptoms emerged as the strongest correlate of overall QoL (19). Female gender and older adolescent age (later puberty) are also prominent factors associated with higher risk. Females with AIS not only represent the majority of patients but also exhibit tighter connectivity within anxiety-depression symptom networks, with specific symptoms like irritability and uncontrollable worry being more salient (2). Within brace-treated cohorts, female patients demonstrate significantly greater susceptibility to depression and anxiety compared to males (20). The presence of moderate to severe back pain is another major contributor. A cross-sectional study of Saudi adolescents with AIS identified pain intensity as the most substantial factor negatively associated with health-related QoL, accounting for a significant portion of its variability (4). This relationship is bidirectional, as psychological makeup, including poor self-image and depression, has been shown to be associated with the development of back pain in adulthood among those with AIS (5).

Environmental and psychosocial factors further modulate the association with psychological distress. A weak social support system, poor family functioning, and significant academic pressure may exacerbate psychological distress. The experience of parents is also telling; a systematic review found that parents of children with AIS face challenges related to information needs, treatment concerns (especially regarding surgery), and negative impacts on their own psychological well-being, which can affect the family environment (21). Notably, the co-occurrence of AIS with GD presents an extreme example of compounded psychosocial risk. Although the co-diagnosis is rare (0.32% of AIS patients in one database), adolescents with both AIS and GD exhibit substantially higher rates of psychological comorbidities—including depression (22.2% vs. 3.6%), anxiety (79.0% vs. 25.9%), and suicidal ideation (36.2% vs. 3.8%)—compared to those with AIS alone (3). This underscores the critical importance of considering intersecting identities and stressors. Self-perceived body image disturbance may represent one pathway linking spinal deformity to internalizing disorders. Studies using instruments like the SRS-22 questionnaire consistently show that the Self-Image domain is often one of the most affected, and lower scores in this domain correlate with greater psychological distress (1, 22). However, it should be noted that SRS-22 self-image scores reflect subjective dissatisfaction with appearance, which is distinct from and not equivalent to a clinical diagnosis of depression or body dysmorphic disorder. In summary, the high prevalence of anxiety and depression in AIS arises from a complex interplay of biomechanical severity, treatment-related stressors, demographic vulnerabilities, pain, and socio-environmental contexts, necessitating a holistic, screening-based approach to patient care.

### 2.2 Impact of comorbidity on disease progression and prognosis

The coexistence of anxiety and depression is significantly associated with the disease trajectory and prognosis in AIS, primarily by diminishing adherence to conservative treatments and exacerbating pain and functional impairment. A study focusing on the psychosocial effects of brace treatment using the EuroQoL 5-dimension (EQ-5D) found that female AIS patients undergoing bracing were significantly more susceptible to depression and anxiety, and these psychological states were strongly correlated with poorer health-related QoL (20). This suggests that the psychological burden of bracing, potentially related to body image concerns and discomfort, can create a barrier to consistent wear, indirectly risking poorer control of curve progression. Furthermore, a narrative review of the literature confirms that brace treatment itself is associated with elevated anxiety levels in AIS patients (14). The challenge of adherence is compounded in specific populations; for instance, patients with concurrent AIS and GD exhibit substantially higher rates of anxiety and depression, and bracing is noted to be particularly challenging for their mental health, potentially leading to even lower compliance (3). This interplay suggests a pathway where untreated psychological distress may undermine the foundational non-surgical management of the spinal deformity.

The relationship between negative emotional states and pain perception in AIS is bidirectional and reinforcing, often leading to a debilitating cycle of increased pain complaints and greater functional limitations. A multidimensional clinical profile study of Saudi adolescents with AIS identified pain intensity as the single most substantial factor associated with reduced QoL, even more so than physical activity levels (4). This highlights the central role of pain in the patient experience. Psychological factors are deeply intertwined with this pain experience. Research has shown that the fear of movement, or kinesiophobia, as measured by the Tampa Scale of Kinesiophobia (TSK), is significantly correlated with depression and QoL scores in AIS patients (6). This fear may lead to activity avoidance, deconditioning, and heightened pain sensitivity. The connection is further evidenced by a multivariate logistic regression analysis which identified anxiety, along with age and school absenteeism, as independent predictors of belonging to a high-level pain group among adolescents and young adults with idiopathic scoliosis (23). This creates a “pain-emotion-dysfunction”feedback loop: pain may fuel anxiety and depression, which in turn may lower pain thresholds and promote fear-avoidance behaviors, leading to increased disability. This cycle not only diminishes current QoL but may also have long-term implications for pain chronicity.

Preoperative anxiety and depression levels serve as important psychological factors associated with postoperative outcomes, including pain control, functional recovery speed, and overall satisfaction following surgical intervention. A retrospective study specifically investigating the impact of a preoperative MHD on pain management found that AIS patients with conditions like anxiety or depression experienced significantly greater postoperative pain scores, required more patient-controlled analgesia during their inpatient stay, and were more likely to still be using opioid pain medication at their first follow-up compared to controls without MHDs (24). This indicates that psychological state is directly associated with the postoperative pain experience and analgesic requirements. Furthermore, the presence of these disorders is associated with broader recovery metrics. An analysis of nationwide inpatient data demonstrated that AIS patients with a concomitant diagnosis of major depressive disorder or generalized anxiety disorder had increased odds for a longer hospital length of stay after posterior spinal fusion, even after controlling for other comorbidities (9). While not directly affecting total cost in that study, a prolonged stay often reflects more complex recovery, including challenges with pain management and mobilization. The predictive value of mental health extends to self-image outcomes after surgery; a study on self-image following posterior spinal fusion found that anxiety/depression was an independent negative predictor of self-image scores in both adolescent and young adult idiopathic scoliosis patients (22). Finally, the validity of patient-reported outcome measures like the Patient-Reported Outcomes Measurement Information System (PROMIS) computer adaptive testing confirms strong correlations between legacy mental health domains and PROMIS depressive symptoms and anxiety scores, reinforcing that these psychological constructs are reliably measurable and critically relevant to the overall surgical outcome profile in AIS (25). Therefore, preoperative psychological assessment is not merely about identifying distress but is a vital prognostic tool for anticipating and potentially mitigating suboptimal recovery trajectories.

## 3 Psychosocial stress and cognitive behavioral model

### 3.1 The core role of body image disturbance and social anxiety

The visible physical alterations caused by AIS, such as trunk asymmetry, rib hump, and perceived height discrepancies, constitute the most direct psychosocial stressors associated with body image dissatisfaction and social anxiety in adolescents. Research utilizing validated instruments like the Body Image Disturbance Questionnaire-Scoliosis (BIDQ-S) confirms that these appearance concerns are a central feature of the AIS experience, with studies showing significant correlations between body image disturbance scores and reduced QoL measures (26, 27). The internalization of societal beauty standards, as measured by tools like the Sociocultural Attitudes Towards Appearance Questionnaire (SATAQ-3), further exacerbates this dissatisfaction, particularly in girls undergoing brace treatment, highlighting the significant interaction between physical deformity and cultural pressures (28). This preoccupation with appearance is not merely superficial; it manifests as a specific cognitive attentional bias. Studies using computerized tasks reveal that girls with AIS exhibit faster reaction times and higher accuracy when processing images of disease-relevant body parts (e.g., shoulders and backs) compared to controls, indicating a heightened, automatic focus on their perceived physical flaws (29). This hypervigilance towards the body’s appearance is closely linked with social anxiety. Patients frequently report avoiding activities like swimming, changing clothes in locker rooms, or wearing certain types of clothing due to intense fear of negative evaluation, mockery, or rejection by peers (18). This behavioral avoidance may contribute to social withdrawal and increased feelings of loneliness, creating a fertile ground for the development or exacerbation of depressive symptoms. A national survey in the United States found that over half of adolescent scoliosis patients report a psychiatric comorbidity, with anxiety and negative body image being among the most prevalent conditions, often diagnosed years after the initial scoliosis diagnosis (1). Screening in clinical settings supports this link, with one study finding that 59% of a pre-operative AIS cohort warranted further assessment for a potential diagnosis of social anxiety (30).

The connection between these visible somatic changes and profound emotional distress is critically associated with maladaptive cognitive processes. Catastrophic thinking about the disease—such as beliefs that “my body is deformed” or “I will be disabled in the future”—acts as a key cognitive bridge. This is reflected in the significant correlations found between body image disturbance scores and measures of anxiety and depression (31). The psychological burden is particularly acute during conservative treatments like bracing. The brace itself, while intended to correct the curve, can become a new source of stigma and a constant physical reminder of the condition, potentially linked to decreased self-esteem, appearance anxiety, and further social isolation, which in turn may negatively impact treatment compliance (18). Real-time monitoring via mobile health apps has captured this dynamic, showing that a significant portion of clinical alerts from braced adolescents are related to emotional distress and social challenges (32). This cascade—from visible deformity to body image preoccupation, social avoidance, and catastrophic cognitions—may establish a self-reinforcing cycle that is associated with a significantly elevated risk for comorbid anxiety and depressive disorders. Consequently, interventions that target these cognitive factors, such as CBT, are increasingly recognized as crucial components of holistic care. CBT can help patients challenge and restructure these catastrophic beliefs, develop healthier coping strategies for social situations, and improve overall psychological well-being and treatment adherence (33, 34). The long-term implications are substantial, with evidence suggesting that AIS and its treatment may affect sexual health, intimacy, and sustained body image dissatisfaction into adulthood, underscoring the enduring impact of these early psychosocial stressors (35).

### 3.2 Illness uncertainty and treatment-related stress

The idiopathic nature and unpredictable progression of AIS generate profound and chronic illness uncertainty for patients and their families, serving as a significant source of psychological distress. A qualitative study applying Uncertainty Management Theory (UMT) to individuals diagnosed with scoliosis in adolescence identified key sources of uncertainty spanning medical, financial, social, and career domains (36). Participants appraised this pervasive uncertainty as either negative or neutral, and their management strategies varied accordingly; some sought to reduce uncertainty by seeking social support or disclosing their condition, while others maintained it by avoiding medical information or adopting a “wait-and-see” approach, and some adapted through reframing, self-care, or religion (36). This chronic uncertainty is compounded by the stress associated with the primary treatment modalities, namely bracing and surgery. Brace treatment itself constitutes a distinct and significant stressor. Studies utilizing validated tools like the Bad Sobernheim Stress Questionnaire-Brace (BSSQ-Brace) consistently show that adolescents undergoing brace treatment experience considerable stress, which negatively impacts their QoL (37, 38). This brace-related stress is strongly linked to worsened emotional and social functioning, as well as a poorer perception of trunk appearance (37). The stress may stem from the physical discomfort, restrictions on mobility and social activities, and concerns about self-image imposed by the brace (39). Longitudinal research indicates that lower pre-bracing health-related QoL is independently associated with increased psychological stress during brace treatment, suggesting that early psychological screening can identify high-risk patients (11). While stress levels may be highest at the initiation of treatment, longer brace duration is associated with lower stress levels and better QoL, likely due to physical and psychological adaptation over time (40). The validation of the BSSQ-Brace in multiple languages, including Korean, Persian, Greek, and Turkish, underscores the universality of this treatment-related stress and the need for cross-culturally relevant assessment tools (41–44).

For patients requiring surgical intervention, the stress profile shifts to fears surrounding the procedure itself, including acute postoperative pain, potential complications, and anxiety about long-term outcomes. The extensive nature of scoliosis surgery presents a major challenge, with severe postoperative pain potentially leading to negative psychological and physical impacts if not managed effectively (45). Rare but distressing complications, such as post-operative Horner’s syndrome, can further contribute to psychological distress (46). Furthermore, surgical correction alters spinal biomechanics, which can induce new sources of mechanical stress and potential long-term physical complications requiring additional management (47, 48). The psychological burden is not limited to the treatment phase but extends to long-term outcomes, with systematic reviews indicating that AIS treatment may adversely affect long-term sexual health, body image, and psychological well-being compared to healthy controls, with brace-treated patients potentially facing greater challenges in these domains (35). This underscores the necessity of a holistic, patient-centered approach that addresses both the physical correction of the deformity and the multifaceted psychological stress arising from illness uncertainty and the demands of treatment.

## 4 The important role of pain in psychophysiological interaction

### 4.1 AIS-related pain characteristics and emotional associations

Back pain is a common but complex symptom in AIS, with a prevalence significantly higher than in the general adolescent population (49). The nature of this pain is predominantly mechanical, often associated with the location of the major coronal curve, with lumbar curves frequently linked to lumbar pain (49, 50). However, the relationship between pain intensity and the magnitude of spinal deformity, such as the Cobb angle, is not absolute or linear (49, 50). Instead, the experience of pain in AIS is multifactorial, heavily influenced by a complex interplay of biomechanical, biochemical, and, most notably, psychological factors (50, 51). For instance, facet joint osteoarthritis, which is associated with axial rotational deformity and kyphotic intervertebral segments, has been directly correlated with increased back pain intensity (52). Post-surgically, while spinal morphology correction generally improves pain, factors like greater residual Cobb angle differences between curves or residual thoracic deformity can be associated with higher pain severity at follow-up (53). This underscores that pain in AIS cannot be predicted by radiographic parameters alone.

The bidirectional relationship between pain and psychological distress is a central feature of AIS. Anxiety significantly influences pain perception; patients with higher baseline anxiety are more likely to follow a postoperative pain trajectory characterized by moderate acute pain with incomplete resolution at one year, resulting in worse QoL (54). Preoperative MHDs are independently associated with greater postoperative pain and increased demands for patient-controlled analgesia (24). Conversely, chronic pain acts as a persistent stressor that may contribute to depressive states. This is supported by the strong association between pain intensity and poorer self-reported mental health scores in AIS patients (4, 5). A critical psychological construct in this dynamic is pain catastrophizing—a pattern of negative, exaggerated thoughts and feelings about pain. Higher levels of pain catastrophizing are prevalent in AIS and are strongly correlated with increased pain intensity, greater preoperative anxiety, kinesiophobia (fear of movement), and poorer scores across all health-related QoL domains, including mental health (6, 50, 55). Catastrophizing is not only associated with the subjective pain experience but may also be linked to the relationship between deformity severity and pain intensity (50). It is a significant correlate of persistent postsurgical pain and is associated with specific gene expression profiles, highlighting a psychobiological pathway (56).

Pain-related functional limitations may contribute to a cycle that exacerbates psychological distress. Back pain often is associated with disability, reducing patients’ ability to engage in prolonged sitting, sports, and other physical activities (51). This restriction may deprive a crucial avenue for mitigating negative emotions, as physical activity is known to buffer against anxiety and depression. The resulting decrease in activity and social participation can lead to isolation, further intensifying feelings of helplessness and depression (43). This cycle is particularly detrimental for adolescents, for whom peer interaction and physical competence are developmentally vital. The functional impairment and associated psychological burden are reflected in validated outcome measures, where pain interference and behavior scores show strong negative correlations with overall QoL (4, 25). Consequently, pain in AIS is not merely a somatic symptom but a core driver of a biopsychosocial predicament where mechanical dysfunction, emotional vulnerability, and behavioral avoidance interact to sustain suffering and disability.

### 4.2 Neuropsychological mechanisms of pain processing

Functional neuroimaging studies suggest that chronic pain and mood disorders may share overlapping neural circuits, including the anterior cingulate cortex, insula, and prefrontal cortex, providing a neurobiological basis for their comorbidity. Research in other chronic pain conditions, such as neuropathic pain and fibromyalgia, has demonstrated that these shared brain regions are critically involved in both nociceptive and affective processing. For instance, in a mouse model of chronic neuropathic pain, the infralimbic-basolateral amygdala circuit was specifically implicated in the development of depression-like, but not anxiety-like, behaviors, highlighting a selective neural pathway for pain-emotion comorbidity (57). Similarly, studies on fibromyalgia syndrome have shown that long-term patients exhibit widespread reductions in white matter-gray matter functional connectivity, particularly involving tracts like the left uncinate fasciculus that link limbic regions (crucial for emotion) with cortical areas involved in pain modulation (58). Furthermore, glutamatergic neurons in the ventral hippocampal CA1 region have been identified as a critical hub, with distinct downstream projections to areas like the nucleus accumbens and lateral septum differentially regulating nociceptive hypersensitivity and depression-like behaviors in a comorbidity model (59). These findings underscore that maladaptive neuroplasticity within these shared circuits—often driven by persistent nociceptive input and neuroinflammation—can lead to the co-occurrence of pain and negative affect. This shared circuitry may explain why chronic pain states are frequently linked to conditions like anxiety and depression, as the neural substrates for processing aversive sensory and emotional experiences are fundamentally intertwined. It is important to note, however, that these findings derive from animal models and other chronic pain populations; direct evidence from adolescents with AIS is currently unavailable, so these mechanisms remain proposed or candidate explanations.

In patients with AIS, long-term pain may lead to adaptive (or maladaptive) functional and structural changes in these brain regions, resulting in diminished emotional regulation capacity and heightened reactivity to negative stimuli. Direct neuroimaging evidence in AIS is limited; however, extensive clinical research provides indirect evidence linking chronic pain to psychological distress and altered pain processing mechanisms. Specifically, a significant proportion of AIS patients report chronic back pain, which is multifactorial and strongly associated with psychological constructs such as pain catastrophizing—a pattern of negative cognitive-emotional responses to pain (50). This psychological factor not only correlates with higher pain intensity but may also influence the relationship between deformity severity and pain perception, acting as a correlate that amplifies the sensory and emotional experience of pain (50). The persistence of pain, particularly after surgical interventions like posterior spinal fusion, can evolve into chronic postsurgical pain, which is associated with functional disability and reduced QoL (60). Studies investigating recovery trajectories after spinal fusion surgery in adolescents have identified that pre-surgical pain intensity and pain catastrophizing are significant correlates of poorer recovery in health-related QoL and pain outcomes (61). This suggests that pre-existing maladaptive psychological responses to pain may prime the central nervous system, potentially reflecting or driving the functional alterations in brain circuits responsible for emotion regulation. Furthermore, factors like kinesiophobia (fear of movement due to pain) in AIS patients show a positive correlation with pain intensity, indicating that pain may be linked to behavioral and cognitive adaptations that further restrict activity and may reinforce negative emotional states (62). The transition from acute to chronic pain states in AIS may involve similar central sensitization and neuroplastic changes observed in other chronic pain populations, where prolonged nociceptive input alters the function of the anterior cingulate cortex and prefrontal regions, impairing top-down pain inhibitory controls and emotional regulation. This is supported by findings that adolescents with higher pre-operative pain catastrophizing have lower self-reported scores across multiple domains, including mental health, both before and even two years after surgery, indicating a lasting impact on psychological well-being (55). Therefore, in AIS, chronic pain likely acts as a persistent stressor that may contribute to maladaptive plasticity within shared brain networks, reducing resilience, enhancing sensitivity to negative stimuli, and creating a feedback loop that may foster the development and maintenance of comorbid anxiety and depression. It is crucial to emphasize that no direct evidence has yet demonstrated these neural circuit changes specifically in adolescents with AIS; the mechanisms proposed here are candidate mechanisms that require direct human AIS investigation to be validated.

## 5 Neuroendocrine and immune-inflammatory pathways

### 5.1 Hypothalamic-pituitary-adrenal axis dysfunction

Chronic psychosocial stress and/or somatic pain can be associated with hyperactivation or dysregulation of the Hypothalamic-Pituitary-Adrenal (HPA) axis, resulting in abnormal cortisol secretion, which is closely linked to the pathogenesis of anxiety and depressive disorders (63). In the context of AIS, the condition itself and its associated treatments constitute significant chronic stressors. The spinal deformity and the resulting postural abnormalities can be associated with persistent bodily pain and discomfort, which are established somatic stressors capable of activating the HPA axis (64). Furthermore, the psychosocial burden of AIS is substantial. Concerns about body image due to the visible rib hump and trunk asymmetry, coupled with the physical limitations imposed by the deformity, can induce significant psychological distress (43). This distress is often compounded by the treatment regimen, particularly brace wear. Bracing, while a primary non-surgical treatment to halt curve progression, introduces its own set of stressors, including physical discomfort, restricted mobility, social embarrassment, and feelings of isolation, collectively termed brace-induced stress (43). Studies utilizing validated instruments like the BSSQ have quantified medium to high levels of stress specifically related to brace wear in AIS patients, highlighting the chronic nature of this treatment-related psychological burden (43). This confluence of chronic physical pain from the deformity and chronic psychosocial stress from the condition and its management creates a sustained demand on the body’s stress response systems. Preliminary research suggests that such prolonged stress exposure in vulnerable individuals may be a key factor in the observed neuropsychological alterations associated with AIS, potentially mediated through HPA axis dysregulation (63). The concept of AIS as a potential “conversion reaction to stress” implicates stress-related neurobiological mechanisms, including HPA axis function, in its pathogenesis and persistence, providing a theoretical link to comorbid emotional symptoms (63).

Emerging evidence from broader biological studies on AIS provides indirect support for the involvement of stress pathways. Research on the molecular pathology of scoliotic intervertebral discs has revealed a distinct pro-inflammatory and pro-oxidative stress state compared to normal discs (65). Proteomic analyses show upregulation of inflammatory markers like complement component 3 (C3) and oxidative stress response proteins such as peroxiredoxins and catalase in scoliotic discs (65). Systemic oxidative stress and neuroinflammation are increasingly recognized as components of AIS pathogenesis, as demonstrated in genetic models where defects in proteins like Scospondin are sufficient to induce these responses alongside spinal deformity (66). Chronic inflammation and oxidative stress are known to interact bidirectionally with HPA axis function; pro-inflammatory cytokines can stimulate the HPA axis, while glucocorticoids modulate inflammatory responses. Therefore, the localized and potentially systemic inflammatory milieu in AIS could contribute to or result from HPA axis alterations. Furthermore, biomechanical studies indicate that the scoliotic spine is under abnormal and asymmetrical mechanical stress. Finite element analyses demonstrate that scoliotic vertebrae and discs experience significantly higher and more heterogeneously concentrated stresses during activities like walking or exposure to vibration compared to a normal spine (64, 67). This chronic, abnormal biomechanical loading represents a form of physical stress that may not only drive curve progression but also contribute to a systemic stress response, potentially influencing neuroendocrine pathways including the HPA axis. The altered sagittal morphology in different AIS types (e.g., Roussouly classifications) further influences spinal biomechanics and load distribution, which could modulate the magnitude of this physical stress signal (68).

Preliminary research hints that some AIS patients may exhibit altered HPA axis reactivity, which could serve as a crucial physiological bridge connecting disease-related stress with emotional symptoms, though more targeted studies in the AIS population are needed for confirmation (63). The integrative framework proposing AIS involves interactions between atypical neuroanatomy, stress processes, and altered body representation positions HPA axis function as a plausible factor (63). The chronic stressors inherent to AIS—encompassing deformity-related pain, brace-induced discomfort, and psychosocial distress—are classic activators of the HPA axis. The downstream effects of a dysregulated HPA axis, such as abnormal cortisol rhythms (e.g., elevated evening cortisol or flattened diurnal slope), are robustly linked to the development and maintenance of anxiety and depression. Therefore, it is biologically plausible that the high prevalence of internalizing symptoms in AIS is partly mediated by HPA axis dysfunction. However, direct evidence measuring cortisol levels, diurnal rhythms, or stress reactivity (e.g., via Trier Social Stress Test) in AIS cohorts compared to healthy controls is currently lacking. Consequently, the role of HPA axis dysfunction in explaining anxiety or depression in adolescents with AIS has not been demonstrated as an established mechanism. Future interdisciplinary research integrating clinical psychology, endocrinology, and biomechanics is essential to directly test this hypothesis. Such studies could measure salivary cortisol profiles, inflammatory biomarkers, and psychological stress (using tools like the BSSQ) in AIS patients with and without comorbid anxiety/depression. This would clarify whether HPA axis dysregulation is a shared mechanism contributing to both spinal deformity progression and emotional comorbidity, paving the way for integrated interventions that address both the physiological stress response and psychological well-being, as suggested by the potential benefits of stress-modulation strategies in AIS management (63).

### 5.2 Involvement of the inflammatory-immune system

The “inflammatory hypothesis” is a key pathophysiological mechanism for depression, where chronic stress and pain can promote the release of peripheral pro-inflammatory cytokines such as interleukin (IL)-6 and tumor necrosis factor-alpha (TNF-α) (69). These cytokines can cross the blood-brain barrier to affect the central nervous system, inducing neuroinflammation, disrupting neurotransmitter metabolism and neuroplasticity, and thereby contributing to the onset of depression and anxiety (69, 70). This mechanistic link is supported by evidence across various conditions, including chronic inflammatory states like obesity, where depression and perceived stress are strongly associated with elevated serum levels of pro-inflammatory markers such as intracellular adhesion molecule-1 (ICAM-1), E-selectin, and C-reactive protein (CRP) (71). In the context of AIS, emerging evidence suggests that this condition is not merely a structural deformity but is associated with a state of chronic low-grade inflammation. A comprehensive review indicates that AIS involves altered systemic immunity, bone metabolism, and paraspinal muscle structure, driven by dysregulated cytokine activity involving IL-6, IL-1β, TNF-α, and IL-17 (72). This inflammatory milieu promotes pathological processes such as osteoclastogenesis, extracellular matrix degradation, and reduced bone mineral density, which are central to curve progression (72). Furthermore, local tissue responses, such as fibrosis and macrophage imbalance in paraspinal muscles, are consistent with persistent inflammatory signaling (72). The potential link between this systemic and local inflammation in AIS and the development of mood disorders represents a novel and supporting direction for exploration. Clinical studies have begun to correlate inflammatory markers with AIS severity. For instance, one pilot study found significant correlations between spinal curvature severity (Cobb’s angle and a composite Scoliosis Score) and specific inflammatory blood parameters, such as beta-2 and gamma globulins, suggesting an interplay between spinal deformity and inflammatory processes (73). This low-grade inflammatory state in AIS may be further modulated by factors such as gut microbiota dysbiosis, which can contribute to endotoxemia and immune activation, potentially affecting systemic inflammation and, by extension, central nervous system function (72). The involvement of inflammatory pathways is also evident at the tissue level in AIS. Research on intervertebral disc degeneration, a common comorbidity, shows that cytokine-induced inflammation (e.g., by IL-1β, TNF-α, IL-6) alters cellular glycosylation and promotes a catabolic state in human nucleus pulposus cells, which could contribute to pain and structural degeneration (74). Similarly, studies on annulus fibrosus cells from AIS patients demonstrate that an inflammatory milieu (e.g., IL-1β stimulation) has a stronger impact on inducing a degenerative phenotype than physiological mechanical stress alone, highlighting the significant role of inflammation in tissue pathology (75). The complement system, another arm of innate immunity, is also activated in disc degeneration associated with AIS, with terminal complement complex (TCC) deposition correlating with degeneration severity and being modulated by pro-inflammatory factors like IL-1β and cathepsin D (76, 77). This local spinal inflammation could be a source of chronic pain, which itself is a significant stressor and trigger for peripheral inflammatory cytokine release, potentially creating a feedback loop that exacerbates both the musculoskeletal condition and risk for mood disorders (69, 78). The hypothesis that AIS-related inflammation might contribute to anxiety and depression is further supported by analogous mechanisms observed in other chronic conditions. For example, in diseases like Crohn’s disease, anxiety and depression are linked to increased aortic stiffness, with chronic inflammation proposed as a mediating factor in a brain-gut-vascular axis (79). In fibromyalgia, comorbid anxiety and depression are associated with altered cerebellar myelination, potentially reflecting long-term consequences of low-grade inflammation (80). Furthermore, interventions that reduce inflammation, such as electroacupuncture, have been shown to mitigate depression- and anxiety-like behaviors in animal models of chronic neuroinflammation by modulating pathways like brain-derived neurotrophic factor (BDNF) and NF-κB (81). Similarly, anti-inflammatory dietary components or compounds like β-caryophyllene can improve cognitive deficits and depressive-like behaviors in rodent models, partly through reducing inflammatory cytokine levels (82). In AIS, the management of postoperative inflammation also provides indirect support for this interplay. The use of multiple-dose tranexamic acid in AIS surgery not only reduces blood loss but also significantly inhibits the postoperative inflammatory response, as measured by lower levels of IL-6 and CRP (83). While not directly measuring mood outcomes, this suggests that controlling the inflammatory surge associated with surgical trauma could have broader systemic benefits. Therefore, the potential mild systemic inflammatory state in AIS, driven by immune dysregulation, local tissue damage, and possibly gut-brain axis alterations, presents a plausible biological substrate that could interact with psychological stress to increase vulnerability to anxiety and depression (72, 73). However, it is important to recognize that direct evidence demonstrating that immune-inflammatory dysregulation is an established mechanism explaining anxiety or depression specifically in adolescents with AIS is currently lacking. Investigating specific inflammatory biomarkers (e.g., IL-6, TNF-α, CRP) in AIS patients with and without comorbid mood symptoms, and exploring how anti-inflammatory strategies might impact both curve progression and emotional well-being, are crucial future research directions to elucidate this psychophysiological interaction.

## 6 Integrated perspective on assessment and diagnosis

### 6.1 Application of psychological assessment tools

The systematic application of validated psychological screening instruments is important for identifying individuals with AIS who may be at elevated risk for comorbid anxiety and depression. It must be clearly delineated, however, that tools such as the Patient Health Questionnaire-9 (PHQ-9) for depression and the Generalized Anxiety Disorder-7 (GAD-7) for anxiety are screening instruments, not diagnostic tests (2). Their role is to efficiently identify patients who warrant further diagnostic evaluation; a positive screen does not confirm a diagnosis but indicates the need for a more comprehensive clinical assessment by a qualified mental health professional. Their utility in AIS populations is supported by network analyses that have identified central symptoms like “sad mood” (PHQ2) and “excessive worry” (GAD3) as potential intervention targets (2). The importance of such screening is underscored by studies showing that over half of adolescent scoliosis patients report a psychiatric comorbidity, with depression and anxiety being the most prevalent, often diagnosed years after the initial spinal diagnosis (1). Routine mental health screening in clinical settings has revealed that a significant proportion of AIS patients warrant further assessment for depression and social anxiety, supporting its consideration for integration into standard care (30).

Beyond generic mental health screening, disease-specific instruments such as the SRS-22 questionnaire serve a different purpose: they assess condition-specific QoL, including mental health, self-image, pain, and function. For example, the SRS-22 mental health domain provides valuable insights but should not be used as a stand-alone screening tool for clinical depression (84). Research indicates that scores on the SRS-22 mental health domain correlate with dedicated depression screening tools; for instance, patients scoring below 3.6 on this domain are at increased risk for moderate-to-severe depression and should be considered for further evaluation with a dedicated screening tool like the PHQ-9, followed by a diagnostic interview if indicated (85).

A comprehensive psychological assessment for AIS may also extend beyond core mood symptoms to capture other facets of psychological impact. This includes a specific focus on body image disturbance, a prevalent issue where negative self-perception is frequently reported by patients (1). Instruments adapted to assess body image concerns are therefore essential. Additionally, evaluating cognitive-affective responses like pain catastrophizing is crucial, as preoperative helplessness and pain catastrophizing have been identified as significant predictors of persistent postoperative pain (56). Similarly, assessing kinesiophobia (fear of movement) is important, as it correlates with pain intensity and can impact quality of life and rehabilitation adherence (6). Evaluating social function and overall QoL completes the psychological profile. Studies consistently show that psychological factors, including depressive symptoms and anxiety, are strong determinants of QoL outcomes in AIS, sometimes outweighing the influence of curve magnitude itself (19, 62).

For treatment monitoring, efficient systems such as PROMIS computerized adaptive tests can reduce administrative burden while effectively measuring domains like anxiety, depressive symptoms, and pain interference over time (86). These are suitable for tracking changes in response to interventions but are not intended for initial diagnosis. In summary, a comprehensive assessment for AIS should employ a multi-tool approach with clearly differentiated purposes: screening instruments (e.g., PHQ-9, GAD-7) identify individuals at risk and should be followed by a diagnostic interview for positive screens; condition-specific instruments (e.g., SRS-22) assess health-related QoL; and tools like PROMIS can monitor treatment progress. Until guideline-level evidence recommends universal screening for all AIS patients, a more measured approach—such as routine psychosocial screening that should be considered—is appropriate.

### 6.2 Construction of a multidisciplinary assessment team

-The ideal assessment model involves a multidisciplinary team comprising orthopedic/spine surgeons, psychologists/psychiatrists, rehabilitation therapists, and nurses working collaboratively.

The construction of a multidisciplinary assessment team is paramount for the comprehensive evaluation of AIS, particularly given the high prevalence of comorbid psychological conditions such as anxiety and depression. An ideal model integrates the expertise of orthopedic or spine surgeons, psychologists or psychiatrists, rehabilitation therapists, and nurses to holistically address the biopsychosocial needs of the patient (1). This collaborative approach is essential because AIS is not merely a structural deformity but a condition with significant psychological ramifications. Studies consistently report elevated rates of MHDs in this population, with clinical depression and anxiety being the most common psychiatric comorbidities, often diagnosed years after the initial scoliosis diagnosis (1). Furthermore, the presence of a preoperative MHD in AIS patients undergoing posterior spinal fusion is associated with poorer postoperative outcomes, including increased pain scores, greater demands for patient-controlled analgesia, and higher rates of continued opioid use at follow-up (24). This underscores the necessity for psychological expertise within the assessment team to identify and address these issues preemptively. The team must function in a coordinated manner, with the orthopedic surgeon providing critical data on spinal deformity severity, curve progression, and surgical indications, while the mental health professional conducts standardized psychological screening and, when positive, a diagnostic assessment. For instance, routine screening in a spinal deformity center revealed that 18% of AIS patients warranted further assessment for potential depression and 59% for social anxiety, highlighting the scale of unmet mental health needs (30). The rehabilitation therapist contributes by assessing functional limitations, pain-related kinesiophobia, and physical activity levels, which are integral to QoL (4). Nurses play a crucial role in patient education, monitoring treatment adherence, and providing continuous psychosocial support throughout the care continuum. The integration of these diverse perspectives ensures that the assessment moves beyond radiographic parameters to encompass the patient’s lived experience, including stressors related to bracing, body image disturbances, and social interactions (8). Effective team function relies on shared communication protocols and the use of integrated assessment tools that capture both physical and psychological domains efficiently, such as the PROMIS computerized adaptive tests, which have demonstrated construct validity for assessing anxiety and depressive symptoms in AIS populations with minimal administrative burden (25). Ultimately, this multidisciplinary framework lays the essential foundation for developing truly individualized, integrated intervention plans that target both the spinal deformity and its psychological sequelae.

- The team needs to integrate findings from imaging, clinical physical examinations, and psychological assessments to gain a comprehensive understanding of the patient’s biopsychosocial status, thereby establishing a foundation for developing personalized intervention plans.

The core function of the multidisciplinary team is the systematic integration of data from imaging studies, clinical physical examinations, and psychological assessments to formulate a holistic biopsychosocial profile of the adolescent with AIS. This integrative analysis is critical because the relationship between deformity severity and psychological distress is not linear; rather, mental health outcomes are influenced by a complex interplay of morphological, perceptual, and psychosocial factors (50). The team begins with standard radiographic evaluations, including Cobb angle measurement and assessment of three-dimensional parameters like axial vertebral rotation and sagittal alignment, which provide objective measures of the structural deformity (87). However, these radiographic findings may best be interpreted in conjunction with clinical examination results, such as angle of trunk rotation, shoulder asymmetry, and functional assessments of mobility and pain (88). Crucially, this physical data set may be combined with standardized psychological assessments. Validated instruments are valuable for this task. The SRS-22/SRS-22r questionnaire remains a key component, assessing domains of pain, function, self-image/appearance, mental health, and satisfaction/dissatisfaction with management (89). Its mental health subscale has shown significant correlations with postoperative self-image and overall satisfaction, demonstrating the link between psychological state and perceived surgical outcomes (89). Furthermore, network analysis of depression and anxiety symptoms in AIS has identified “Excessive worry” (GAD3) and “Sad mood” (PHQ2) as central symptoms across subgroups, offering potential targets for psychological intervention (2). The team may also use condition-specific tools, such as the BSSQ for brace-related stress or the Tampa Scale for Kinesiophobia (TSK) for fear of movement, to capture treatment-specific psychological burdens (6, 38). Integrating these findings reveals critical patterns. For example, research indicates that psychological factors like pain catastrophizing and self-reported mental health status are independent correlates of back pain intensity in AIS, sometimes exerting a greater influence than radiographic severity (50). Similarly, discrepancies between adolescent and parent ratings on health-related QoL questionnaires, particularly in self-image and mental health domains, are associated with higher adolescent psychological symptom levels and can predict poorer adherence to prescribed therapies like exercise (90). This comprehensive profiling allows the team to identify high-risk individuals, such as those with co-occurring gender dysphoria, who exhibit substantially higher rates of anxiety, depression, and suicidal ideation compared to those with AIS alone (3). By consolidating imaging, clinical, and psychological data, the multidisciplinary team can move beyond a one-size-fits-all approach. They can discern whether a patient’s primary barrier to recovery is structural instability, debilitating kinesiophobia, severe body image disturbance, or a combination thereof. This nuanced understanding is the essential prerequisite for crafting a truly personalized intervention plan that may combine surgical correction or bracing with cognitive-behavioral therapy, pain management strategies, family counseling, or targeted psychosocial support, ultimately aiming to improve both physical alignment and mental well-being (33).

## 7 Psychological and social intervention strategies

### 7.1 CBT and acceptance and commitment therapy

CBT is a key component psychological intervention for AIS, aiming to modify maladaptive thought patterns and behaviors associated with the condition. Its primary goal is to help patients identify and correct negative automatic thoughts and beliefs concerning their disease, body image, and future, thereby reducing catastrophic thinking. CBT equips adolescents with behavioral skills to manage pain and navigate challenging social situations, which is crucial given the psychosocial burden of AIS, including concerns about appearance and social withdrawal that can hinder treatment compliance (18). The application of CBT extends across the treatment continuum. For instance, specific cognitive-behavioral support programs for patients and parents have been shown to significantly enhance adherence to brace-wearing, a critical determinant of treatment success (91). In the surgical context, a trial protocol proposes perioperative digital CBT interventions, such as SurgeryPal™, to target psychosocial correlates such as low pain self-efficacy. This intervention is being evaluated for its potential to prevent the transition from acute to chronic postsurgical pain by improving coping strategies, thereby addressing a significant source of long-term distress (92). Furthermore, CBT directly addresses body image dissatisfaction, a core psychological challenge in AIS. Studies utilizing tools like virtual reality to assess body perception have investigated CBT’s role in helping patients reconcile their desired body shape with their actual postoperative appearance, potentially reducing preoperative dissatisfaction and fostering a more positive postoperative self-view (93). The versatility of CBT principles is also evident in innovative delivery methods, such as chatbot-delivered structured psychological interventions (SPI-Bot), which incorporate cognitive and behavioral strategies to provide accessible, real-time support for managing psychological distress in teenagers with AIS (94). This trial is being evaluated to assess feasibility and preliminary effectiveness.

In contrast, a potential future approach is Acceptance and Commitment Therapy (ACT), which offers a value-based framework by focusing on psychological flexibility rather than direct cognitive change. Although the provided references do not explicitly evaluate ACT in AIS, its core processes could theoretically complement existing interventions. ACT would aim to help patients accept the inevitable distress and physical changes associated with scoliosis, such as alterations in trunk appearance or the discomfort of bracing, without futile attempts to eliminate these experiences. Therapy would guide adolescents to clarify their personal values—what is truly important to them beyond their spinal condition—and to commit to actions aligned with those values, even in the presence of pain or self-consciousness. This theoretical process of valued living is intended to reduce the impact of emotional suffering and improve psychological well-being. The concept of reducing experiential avoidance and enhancing meaningful engagement is indirectly supported by findings that psychosocial interventions improve coping and quality of life (18). However, it must be emphasized that ACT should not be presented as an established intervention for AIS based on the current evidence; instead, it warrants future empirical investigation in controlled trials within the AIS population.

The group therapy format is particularly effective for delivering both CBT and ACT interventions to adolescents with AIS. This setting provides a powerful platform for peer support, which can directly counteract feelings of isolation and stigma associated with wearing a brace or having a visible deformity (18). Within a group, adolescents can share experiences, normalize their challenges, and learn from others facing similar situations, which reduces the sense of being uniquely afflicted. Furthermore, the group serves as a safe and structured environment to practice newly acquired social skills and behavioral techniques, such as communicating about their condition with peers or managing anxiety in social settings. The collective experience of working through cognitive restructuring exercises or value-clarification activities can enhance learning and motivation. The importance of social support is underscored by its inclusion as a measured outcome in trials of psychological interventions for AIS; for instance, a pilot randomized controlled trial protocol for SPI-Bot includes social support as a proposed outcome (94). Therefore, group-based psychological therapies not only address individual cognitive and behavioral patterns but also leverage the therapeutic factors inherent in group dynamics, such as universality and instillation of hope, to foster resilience, improve treatment adherence, and enhance overall psychosocial functioning in this population.

### 7.2 Mindfulness-based stress reduction and relaxation training

Mindfulness-based interventions, including formal mindfulness practice and relaxation techniques, represent a potential non-pharmacological approach for managing psychological and physical comorbidities in AIS, but direct evidence from AIS-specific mindfulness trials is lacking. While mindfulness practice encourages non-judgmental awareness of present-moment experiences—theoretically disrupting maladaptive cycles of pain, anxiety, and avoidance—the cited studies do not describe standard mindfulness protocols. Research on biofeedback posture training and surface electromyography (sEMG) reports qualitative benefits such as reduced pain and fatigue, and improved self-confidence and self-image (95), and studies of the Schroth method show changes in paraspinal muscle symmetry during specific exercises (96). However, these findings arise from distinct rehabilitation mechanisms—such as postural control and muscle relaxation—and cannot by themselves establish the effectiveness of mindfulness for anxiety or depression in AIS. Similarly, relaxation techniques like progressive muscle relaxation and diaphragmatic breathing directly downregulate physiological arousal, yet their specific impact on AIS outcomes remains unconfirmed by direct trials. Therefore, mindfulness and relaxation should be presented as possible future interventions requiring dedicated AIS research, rather than as evidence-based components of current care. Integrating them into a biopsychosocial model for AIS holds theoretical promise, but until direct trials demonstrate efficacy, their role remains speculative.

### 7.3 Family intervention and school support

Family intervention and school support are important components of a holistic management strategy for AIS, and have been associated with the psychological well-being and treatment adherence of affected adolescents. Educating parents is paramount, as their understanding and response significantly influence the adolescent’s emotional landscape. Research indicates that a supportive family environment, characterized by appropriate emotional support rather than overprotection or criticism, is crucial for the adolescent’s emotional regulation and overall mental health (10). A qualitative study exploring brace compliance highlighted that family dynamics, including supervision and support, are integral themes affecting treatment success, underscoring the need for parents to be guided on fostering a positive and understanding home atmosphere (97). Furthermore, a cross-sectional study in China found that family functioning in AIS families was often affected, with scores in affective involvement and behavior control indicating areas of strain, suggesting that targeted family interventions could improve these dynamics and, by extension, patient outcomes (98). The process of brace compliance itself is complex and influenced by the family system; a qualitative study identified that extensive lifestyle changes and the need for long-term commitment within the family unit are key themes, emphasizing that parental education may address these practical and emotional challenges to sustain adherence (99). Providing parents with clear, structured information can also alleviate anxiety and improve shared decision-making, as evidenced by resources developed to enhance health literacy before clinical consultations, which help families feel more prepared and involved (100).

Concurrently, creating a supportive school environment is equally vital to mitigate the psychosocial burdens of AIS. Collaboration with educational institutions to educate teachers and peers about AIS can reduce stigma, misunderstanding, and discrimination, thereby fostering a more inclusive atmosphere for the adolescent (101). A qualitative study specifically investigating the school-time experiences of adolescents undergoing brace treatment revealed significant concerns and actual problems related to social interactions and participation, highlighting the critical need for school-based support programs (101). Adolescents often report feeling self-conscious and alienated while wearing a brace, frequently hiding it under clothing due to fear of negative peer feedback, which directly impacts their social engagement and mental health (102). Therefore, school interventions that normalize the condition and promote peer understanding are essential. Moreover, the broader school environment can play a role in prevention and general well-being. A systematic review on physical activity and AIS proposed an integrated “school-family” and “school-sports” approach, suggesting that schools should not only be sites for support but also active partners in promoting spinal health through structured activity programs (103). This aligns with findings that psychosocial challenges, including stress and poor body image, are significant barriers to brace compliance, and supportive environments that extend into the school setting can enhance treatment fidelity (10). Ultimately, a coordinated effort between healthcare providers, families, and schools is necessary to address the multifaceted challenges of AIS, ensuring adolescents receive the comprehensive emotional and social support required for optimal psychological adjustment and treatment success.

## 8 Integration of biomedical and rehabilitation interventions

### 8.1 Pain management and pharmacotherapy

The management of pain in AIS is a key component of comprehensive care, particularly given the frequent association of the condition with spinal pain and its significant impact on QoL (62). Standardized physical therapy and exercise regimens form the foundational, non-pharmacological approach. A meta-analysis has demonstrated that specific spinal exercise therapy is effective not only in improving structural parameters like the Cobb angle and trunk rotation but also in positively impacting pain scores, functional activity, and self-image compared to other non-exercise conservative treatments (12). This underscores the dual role of structured physical activity: managing the physical deformity and its associated discomfort while simultaneously enhancing psychological well-being and patient satisfaction with treatment. The relationship between pain and movement is further complicated by psychological factors such as kinesiophobia, which is a fear of movement due to pain. Studies show that kinesiophobia in AIS patients correlates positively with pain intensity rather than the severity of the spinal deformity itself, highlighting that pain management and reassurance are critical to reducing movement-related fear, improving adherence to therapeutic exercise, and ultimately achieving better rehabilitation outcomes (62). Therefore, a well-structured physiotherapy program may support functional improvement and mood elevation, and can represent a valuable intervention option for AIS-related pain.

The major change for postoperative pain management in AIS surgery has evolved significantly towards multimodal analgesic regimens, which aim to minimize opioid consumption and its associated adverse effects while providing effective analgesia (104). Traditional reliance on high-dose opioids via PCA is being supplanted by protocols that integrate various non-opioid agents. Evidence favors the inclusion of neuraxial techniques (epidural or intrathecal opioids), scheduled acetaminophen, and ketorolac within these protocols (105). For instance, a randomized controlled trial found that preventive (postoperative) administration of intravenous acetaminophen significantly reduced cumulative 24-hour opioid consumption compared to placebo in patients undergoing posterior spinal fusion (106). Other effective components include methadone, which acts as both a mu-opioid agonist and an N-Methyl-D-aspartate antagonist; a protocol using scheduled postoperative methadone resulted in a 45% reduction in total opioid use compared to a PCA-based protocol (107). The role of regional anesthesia is also prominent. Studies comparing continuous epidural analgesia and spinal opioids to PCA show that both neuraxial techniques significantly decrease total parenteral and oral opioid requirements, though all methods provide safe and effective pain relief (108). Furthermore, the use of local anesthetics, such as liposomal bupivacaine administered as an erector spinae plane block or via local infiltration, has been associated with decreased total opioid use, shorter hospital length of stay, and improved early ambulation (109, 110). Enhanced Recovery After Surgery (ERAS) pathways that standardize these multimodal approaches have been shown to successfully reduce both length of stay and overall opioid usage without increasing pain scores or readmission rates (111, 112). The efficacy of these comprehensive strategies is further evidenced by studies showing that increased use of intraoperative non-opioid analgesics (e.g., ketamine, dexmedetomidine, gabapentin) contributes to a reduction in postoperative opioid requirements and earlier hospital discharge (113). While gabapentin’s system-wide effect in reducing opioid use is noted, its impact can vary across individual hospital protocols (114).

A critical practical consideration involves patients who are already receiving psychiatric medications (e.g., selective serotonin reuptake inhibitors (SSRIs), serotonin-norepinephrine reuptake inhibitors (SNRIs), benzodiazepines, antipsychotics, or mood stabilizers) when they undergo bracing, rehabilitation, or surgery. These medications may influence orthopedic treatment planning, perioperative management, and postoperative outcomes. For example, SSRIs and SNRIs can interfere with platelet aggregation, potentially increasing perioperative bleeding risk, and may also interact with multimodal analgesic agents such as tramadol or nonsteroidal anti-inflammatory drugs, raising concerns about serotonin syndrome or altered pain perception (115, 116). Preoperative evaluation should, therefore, include a thorough medication reconciliation to assess these risks and adjust regimens as needed, in consultation with the prescribing psychiatrist and anesthesiologist. Additionally, patients with pre-existing mood or anxiety disorders may have lower pain thresholds, higher postoperative pain scores, or reduced adherence to bracing and physiotherapy regimens. Discontinuation of psychiatric medications during the perioperative period—sometimes due to nil per os status, concerns about sedation, or polypharmacy—can destabilize mood and anxiety, exacerbating pain, prolonging recovery, and reducing participation in physical therapy. To mitigate these risks, it is essential that psychiatric pharmacotherapy is not considered in isolation but as part of a coordinated, multidisciplinary plan involving psychiatry, orthopedic surgery, anesthesia/pain management, and primary care. Early consultation can ensure that medications are continued safely, switched to suitable alternatives when necessary, and monitored for interactions with anesthetic agents and analgesics. Ultimately, the goal is a collaborative, individualized plan developed by orthopedic surgeons, anesthesiologists, and pain specialists to optimize recovery, minimize opioid-related risks, and address the intertwined physical and psychological dimensions of pain in AIS (24, 108).

### 8.2 Potential impact of surgical intervention on psychological state

Successful spinal deformity correction surgery for AIS not only addresses the physical deformity by improving body appearance, balance, and preventing curve progression but also confers positive effects on patients’ self-image, pain, and overall QoL, as measured by patient-reported outcomes. A systematic scoping review of qualitative evidence indicates that while some patients find surgery stressful, many are relieved to have undergone the procedure and are generally happy with the outcome, which positively impacts their self-image and perceptions of appearance (117). A prospective registry-based cohort study demonstrated that at two-year follow-up, fusion surgery for AIS led to clinically relevant improvements in SRS-22r domain scores for function, pain, and self-image, as well as the total score, with a significant reduction in the proportion of patients reporting severe back pain (118). A comparative study of surgically versus conservatively treated AIS patients with curves in the operative range found that at two-year follow-up, the surgical cohort had significantly better SRS-22 self-image, mental health, satisfaction, and total scores, which remained largely unchanged in the non-operative group (119). Long-term follow-up studies, including one with a minimum 40-year follow-up after Harrington rod fusion, have associated successful surgical outcomes with patient-reported QoL, although specific parameters like pelvic incidence-lumbar lordosis mismatch were linked to inferior outcomes (120). Furthermore, a study on adult patients with residual AIS (AdIS) undergoing posterior spinal fusion found that despite increased surgical invasiveness, postoperative SRS-22 scores were comparable to those of adolescent patients, with AdIS patients showing significantly greater improvement in the self-image domain (121). Another study comparing adult idiopathic scoliosis patients of different ages found that while adolescent patients had the highest preoperative self-image scores, the greatest postoperative improvement was observed in older adults, despite more surgical morbidity (22). These findings underscore that successful surgical correction may be associated with durable improvements in pain, self-image, and some patient-reported psychosocial outcomes, but psychological responses vary across patients.

However, it is crucial to fully assess patients’ psychological expectations preoperatively, as unrealistically high expectations for cosmetic improvement can lead to postoperative dissatisfaction. A qualitative study on patients who underwent vertebral body tethering or posterior spinal fusion highlighted that patient priorities for success varied, including pain relief, physical function, appearance, and long-term correction, and emphasized the importance of aligning surgical outcomes with these individual goals to ensure satisfaction (122). The Patient Generated Index (PGI) study revealed that AIS patients and their parents often have different primary concerns and decision regrets regarding surgical treatment, with patients frequently reporting sports and general function as top concerns, while parents emphasized general function and appearance (123). This discrepancy underscores the need for comprehensive preoperative counseling to align expectations. Furthermore, specific surgical factors can influence self-image outcomes. For instance, in patients with Lenke type 1 AIS, residual T1 tilt postoperatively was associated with worse self-image scores, indicating that achieving horizontal shoulder balance is a key cosmetic concern for patients (124). Therefore, managing expectations regarding achievable cosmetic correction is essential. Perioperative psychological support should be considered an integral component of standard care. Evidence suggests the feasibility, acceptability, and favorable associations of psychosocial interventions with outcomes such as postoperative pain, anxiety, engagement in activities, and brace compliance (33). For example, a pilot study of an Internet-based cognitive-behavioral intervention for adolescents and parents during the perioperative period was found to be highly feasible and acceptable (125). Similarly, a randomized controlled trial protocol for a digital perioperative cognitive-behavioral intervention (SurgeryPal™) aims to prevent the transition from acute to chronic postsurgical pain by targeting psychosocial correlates (92). Other supportive modalities have shown benefit; a randomized controlled trial of energy healing therapy resulted in a decrease in patients’ preoperative anxiety (126), and a pilot study on virtual reality sessions prior to postoperative physical therapy significantly reduced pain scores associated with therapy (127). Implementing a structured ERAS pathway for spinal surgery has also been associated with reduced postoperative anxiety and higher treatment satisfaction (128). These interventions highlight the critical role of integrated psychological support in optimizing the surgical experience and overall recovery.

The presence of preoperative MHDs, such as anxiety and depression, significantly influences perioperative pain management, recovery trajectories, and overall outcomes, necessitating tailored approaches. A retrospective database study found that approximately 15.92% of AIS patients had a documented preoperative MHD, and these patients experienced higher total opioid use and greater healthcare utilization costs postoperatively, although complication rates were similar (16). Another study specifically on AIS patients undergoing posterior spinal fusion confirmed that those with a preoperative MHD had greater postoperative pain scores, required more PCA demands during their inpatient stay, and were more likely to be using opioid pain medication at the first follow-up (24). Furthermore, a study using the Nationwide Inpatient Sample reported that AIS patients with either major depressive disorder or generalized anxiety disorder had increased odds of a longer hospital length of stay after posterior spinal fusion (9). Preoperative psychological factors are robust predictors of postoperative pain outcomes. Research has consistently identified preoperative pain severity, anxiety, and pain catastrophizing (including feelings of helplessness) as significant predictors of persistent postoperative pain and poorer QoL trajectories (54, 56, 61). For instance, one study found that 36% of adolescents experienced persistent pain at final follow-up, with preoperative pain severity and helplessness being the most significant predictors (56). Another study identified three distinct pain trajectories after surgery, with membership in the “moderate pain with incomplete resolution” trajectory predicted by higher baseline pain and anxiety (54). Pain catastrophizing is a particularly modifiable correlate; it changes throughout the perioperative period, and higher preoperative levels are associated with increased anxiety, kinesiophobia, and worse preoperative and postoperative patient-reported outcomes in domains like pain, appearance, and mental health (55, 129). A latent class growth analysis on health-related QoL trajectories also found that increased child and parent pain catastrophizing and child anxiety were associated with poorer physical and psychosocial health outcomes post-surgery (130). Therefore, preoperative screening for these psychological factors may be valuable. As suggested by research, patients presenting with pain, helplessness, or anxiety during preoperative appointments may need increased vigilance, possibly with bedside quantitative sensory testing and psychological questionnaires administered by nurses (131). Biobehavioral interventions targeting pain intensity, feelings of helplessness, and anxiety during the preoperative period may help mitigate the transition to chronic postsurgical pain (131). Ultimately, policy and practice changes are essential for the early identification and subsequent intervention for at-risk adolescents to optimize recovery trajectories (56).

## 9 Multidisciplinary integrated intervention models and future prospects

### 9.1 Establishing an integrated “mind-body” care pathway

Future clinical practice may benefit from prioritizing the establishment of a standardized, integrated care pathway spanning from screening and assessment to intervention, ensuring that AIS patients can receive routine evaluation for psychosocial needs. Such a pathway could be designed to seamlessly incorporate patient-reported outcome measures into clinical workflows to efficiently capture the biopsychosocial impact of the condition. Research demonstrates that modern tools like the PROMIS computerized adaptive testing measures offer a rapid and low-burden method for assessing key domains, including anxiety, depressive symptoms, peer relationships, and pain interference, with completion times averaging just over one minute per domain (86). This efficiency starkly contrasts with longer legacy measures like the SRS-22r, supporting the feasibility of integrating comprehensive psychosocial screening into routine clinic visits without significant administrative strain (86). The core of this integrated pathway is the necessary, systematic assessment of psychological well-being at initial presentation and at regular intervals throughout treatment, whether the patient is under observation, undergoing bracing, or being considered for surgery. This is critically important as studies consistently highlight the “silent strain” of AIS and its treatments, particularly bracing, which is associated with negative self-image, low self-esteem, social anxiety, and appearance-related concerns that can severely impact adherence and QoL (18). A standardized pathway ensures these often-overlooked psychosocial effects are not missed, enabling early identification of distress.

Furthermore, the integrated care pathway may explicitly define collaboration nodes and referral mechanisms between psychologists/mental health counselors, rehabilitation therapists, and spine surgeons to facilitate seamless integration of biomedical treatment and psychosocial intervention. The pathway should outline clear triggers for referral to mental health professionals, such as identified elevations in anxiety or depressive symptoms on PROMIS computerized adaptive testing, expressed concerns about body image or social withdrawal, or observed non-compliance with bracing protocols linked to psychological factors (18). Effective interventions exist to support this integration. For instance, systematic reviews indicate that psychosocial interventions involving frequent patient-provider interactions, such as brace compliance monitoring combined with counseling, can significantly improve both the quality and quantity of brace wear (132). Similarly, proactive mental healthcare delivery by nurses following spinal surgery has been shown to improve patient outcomes (132). Specific evidence-based interventions like CBT and structured peer support programs have been identified as beneficial for improving coping mechanisms, psychological well-being, and treatment adherence in braced adolescents (18). The collaborative model extends into long-term and transitional care, as managing AIS is a lifelong process. A multidisciplinary care team, engaging neurosurgeons, orthopedic surgeons, mental health professionals, and rehabilitation specialists, is essential for monitoring patients into adulthood, addressing postoperative complications, new diagnoses, and sustained psychosocial support to optimize health-related QoL (133). This holistic, patient-centered approach, embedded within a clear clinical pathway, ensures that psychological support is not an afterthought but a fundamental component of care delivery, ultimately aiming to improve both physical alignment and mental health outcomes for adolescents with scoliosis.

### 9.2 Application of digital health technologies

The application of digital health technologies in AIS management is rapidly evolving, offering innovative tools to enhance both physical treatment adherence and psychological support. A key development is the creation of mobile health applications designed to monitor brace compliance and patient wellbeing. These apps address the significant challenge of low adherence to conservative treatments like bracing, which is often due to discomfort, mobility restrictions, and associated psychosocial stressors (134). A feasibility study demonstrated that an app-based system for daily self-reporting on brace wear duration, discomfort, emotional distress, and social challenges was acceptable to digitally-engaged adolescents (135). In a clinical utility evaluation, participants reported meeting the recommended brace wear time (≥16 hours/day) on 84.8% of days using a custom app over 90 days (32). This same system incorporated a clinical alarm feature that alerted healthcare providers to potential concerns based on patient input, with the most frequent triggers related to emotional distress (23.1%) and pain (15.6%) (32). This functionality allows for early detection of treatment barriers and prompts timely clinical responses, directly addressing the psychosocial challenges that underlie poor adherence. Furthermore, qualitative research involving stakeholders (patients, caregivers, and providers) highlights the demand for digital platforms that offer accessible, adolescent-friendly education on AIS and provide avenues for peer support and community connection, which are crucial for psychosocial wellbeing (134). These apps can be tailored with gamification elements, such as brace compliance tracking and avatar customization, to increase motivation and engagement, transforming treatment from a passive burden into an interactive process (134). The integration of such mobile applications effectively provides a scalable, accessible, and confidential tool for delivering psychological support modules, such as elements of CBT or mindfulness, directly to AIS patients, thereby bridging a critical gap in holistic care.

Beyond compliance monitoring, digital technologies offer new opportunities for the objective assessment of AIS through advanced sensing and imaging. Wearable sensors, including inertial measurement units, are being used to gain deeper insights into the biomechanical and functional consequences of scoliosis. For instance, research utilizing wearable inertial sensors has demonstrated that individuals with AIS exhibit subtle postural control deficits and employ widespread, diffuse compensatory joint control strategies during dynamic balance tasks, especially under medio-lateral demands, compared to the more localized strategies seen in controls (136). This detailed kinematic data, captured during activities like gait, provides a rich source of information beyond static clinical measurements. Machine learning models, particularly deep convolutional neural networks, have been developed to analyze gait data from wearable sensors, such as hip-knee cyclograms, to accurately predict scoliotic curve progression with high accuracy (92%), outperforming traditional models (137). This approach enables a more dynamic and personalized risk assessment. Concurrently, to reduce reliance on serial radiographs for monitoring, AI-powered applications using smartphone-based 3D surface topography have been developed. These apps can predict Cobb angles with high criterion validity, showing strong correlation with radiographic gold standards (r = 0.922) and high sensitivity and specificity at clinically meaningful thresholds (e.g., 0.962 and 0.960 at 10°) (138). Other studies have validated the use of common mobile devices and computer vision techniques, like human pose estimation, to derive photographic parameters (e.g., shoulder height, trunk tilt) that correlate significantly with conventional radiographic measures, offering a radiation-free method for screening and continuous posture monitoring (139). Deep learning models incorporated into open-platform apps can classify AIS severity, curve type, and even identify progression from a single smartphone photograph of the back with performance comparable to experienced spine surgeons (140). When combined, these technologies—wearable sensors for continuous activity and biomechanical monitoring and smartphone apps for periodic posture/curve assessment—facilitate the collection of real-time, ecologically valid data on both physical state and subjective experience (e.g., via ecological momentary assessment integrated into apps). This multimodal data fusion allows for a much more precise and individualized understanding of the psychophysiological interaction patterns in AIS, such as how pain or emotional distress correlates with specific activity levels or postural deviations, paving the way for truly personalized intervention strategies.

Digital health technologies also extend powerfully into the realms of surgical planning, rehabilitation, and psychological therapy for AIS. In preoperative planning, virtual reality has emerged as a transformative tool. Surgeons using virtual reality to explore interactive 3D anatomical models reconstructed from patient computed tomography (CT) scans have reported significant benefits, including reduced operative time and blood loss, and increased satisfaction compared to traditional 2D image planning (141). Furthermore, 3D-printed spine models based on patient-specific biplanar radiographs are being used to simulate surgery virtually, providing a realistic platform for surgical training and instrument testing (142). Mixed reality systems, such as those using the HoloLens 2, are being developed to overlay virtual 3D spine models onto a patient’s actual anatomy or onto 3D-printed models for enhanced intraoperative navigation, with preliminary studies showing a significantly lower rate of pedicle screw misplacement compared to the freehand technique (143). In the postoperative phase, virtual reality is being investigated as a non-pharmacologic adjunct for recovery. A pilot study found that an immersive virtual reality session prior to each inpatient physical therapy session significantly reduced physical therapy-associated pain scores compared to controls, although it did not significantly alter total opioid usage or length of stay in this small cohort (127). More structured virtual reality-based rehabilitation (VRBR) programs have shown efficacy in randomized trials. Patients assigned to a 6-week VRBR program using a system like Kinect Xbox 360 demonstrated statistically significant improvements in spinal mobility, respiratory muscle strength, physical activity levels, and reductions in kinesiophobia compared to a control group performing home-based exercises (144). Perhaps most innovatively, virtual reality is being applied to address the core psychological issue of body image disturbance in AIS. CBT delivered with the aid of a virtual reality application (“Avatar Scoliosis 3D”) has been used to assess and treat body image dissatisfaction (93). Studies using this technology revealed that patients accurately estimate their body shape pre- and postoperatively but feel significant dissatisfaction preoperatively, which resolves after surgery, irrespective of therapeutic support in the studied cohorts (93). This demonstrates virtual reality’s potential as a powerful tool for both assessment and therapeutic intervention in the psychological dimension of AIS, offering immersive experiences that can help patients visualize outcomes and modify maladaptive perceptions about their bodies.

### 9.3 Future research directions

Prospective longitudinal studies would be valuable to clarify the temporal associations and trajectory of comorbidity between AIS and anxiety/depression. The current body of evidence, while robust in establishing a strong association, is predominantly cross-sectional or retrospective, limiting causal inference (14). For instance, a national survey found that 58% of adolescent scoliosis patients reported a mental health diagnosis, with 76% of these diagnoses occurring after the scoliosis diagnosis, hinting at a potential temporal sequence (1). However, this retrospective data cannot definitively establish whether AIS precipitates mental health disorders or if shared vulnerabilities contribute to both. A narrative review of the literature explicitly calls for more longitudinal studies utilizing accurate psychometric instruments to reveal the current and evolving impact of AIS on adolescent mental health (14). Such studies should track cohorts from diagnosis through adolescence and into young adulthood, capturing critical transitions such as the initiation of bracing, surgical intervention, and skeletal maturity. This would allow researchers to determine if specific treatment phases (e.g., brace wear) act as critical periods for the development of anxiety or depression (14, 20), and to identify the natural history of psychological sequelae in conservatively treated patients (5). Furthermore, longitudinal designs are essential for understanding the bidirectional nature of this relationship, such as how pre-existing subclinical anxiety might affect pain perception and treatment adherence, which in turn could influence curve progression and psychological distress (5, 24). Without this temporal clarity, interventions risk being reactive rather than preventive.

Future research may also benefit from prioritizing mechanistic studies utilizing advanced methodologies from neuroimaging, genetics, and immunology to explore distinct biological markers of psychophysiological interaction within the specific context of AIS. While network analysis has begun to identify central psychological symptoms like “Excessive worry” and “Sad mood” (2), the underlying neurobiological substrates linking spinal deformity to these affective states remain poorly understood. Neuroimaging studies could investigate whether chronic postural adaptations and pain processing in AIS lead to functional or structural changes in brain regions associated with body schema (e.g., parietal cortex), emotional regulation (e.g., amygdala, prefrontal cortex), and pain modulation (e.g., anterior cingulate cortex) (6). Genetic research should move beyond establishing a lack of direct Mendelian randomization causality for broad mental disorders (145) and instead explore polygenic risk scores or gene-environment interactions that might predispose certain AIS patients to comorbid anxiety or depression, particularly in response to stressors like bracing or surgery. Immunological pathways represent another frontier; chronic low-grade inflammation associated with musculoskeletal stress or post-surgical recovery could potentially influence mood-regulating neurotransmitters and contribute to depressive symptoms (146). The goal is to move from correlational clinical observations to identifying objective, measurable biomarkers. For example, research could test whether specific patterns of cortisol secretion (a neuroendocrine marker of stress) or inflammatory cytokines correlate with the severity of kinesiophobia (fear of movement) or depression scores in AIS patients undergoing treatment (6, 146). Identifying such markers would not only deepen the understanding of the pathophysiology but also allow for the stratification of patients into high-risk subgroups for targeted monitoring.

Finally, it would be valuable to design and implement rigorous randomized controlled trials (RCTs) to compare multidisciplinary, integrated intervention models with traditional, single-modality treatments in terms of physical and mental health outcomes and healthcare cost-effectiveness. Current management often siloes orthopedic care from psychological support, despite clear evidence that mental health diagnoses like anxiety and depression are associated with worse postoperative pain, increased analgesic requirements, and longer hospital stays (9, 24). An integrated model would proactively embed psychological screening, using efficient tools like PROMIS computerized adaptive testing or EQ-5D, and intervention within standard scoliosis care pathways (20, 86, 147). RCTs are needed to test specific interventions. For instance, trials could compare standard bracing protocols with bracing protocols supplemented with CBT to address anxiety, body image distress, and kinesiophobia (6, 148). Another trial could evaluate a pre-operative psychological preparation program for surgical candidates with identified mental health risks, measuring its impact on postoperative pain, opioid use, and QoL (24). Furthermore, the efficacy of postoperative supports, such as the use of braces for psychological reassurance in addition to physical stability, warrants investigation through controlled studies (148). These trials must employ comprehensive outcome assessments that capture the full biopsychosocial impact, including validated measures of mental health (e.g., PHQ-9, GAD-7), quality of life (e.g., SRS-22r, PROMIS), functional recovery, and direct/indirect medical costs (25, 90). By demonstrating the clinical and economic value of integrated care, such research can drive necessary changes in healthcare policy and clinical practice, ensuring that the management of AIS addresses the whole patient, not just the spinal curve.

## 10 Critical synthesis and interpretation of the evidence

Taken together, the evidence reviewed here supports several conclusions with differing levels of certainty. Findings that are reasonably well supported directly in AIS include the elevated prevalence of screen-identified anxiety and depressive symptoms relative to healthy peers, the association of greater curve severity, female gender, brace treatment, and pain with increased psychological distress, and the robust relationship between preoperative psychological factors (particularly anxiety and pain catastrophizing) and poorer postoperative pain and recovery outcomes. These associations are derived largely from cross-sectional and retrospective AIS-specific studies, often with sizable samples, and are consistent across multiple research groups and clinical settings. The bidirectional relationship between pain and emotional distress in AIS is likewise well documented at the clinical level, as is the central role of body image disturbance and brace-related stress in shaping the psychosocial experience of affected adolescents.

A second tier of evidence concerns mechanisms and interventions that remain preliminary or depend mainly on indirect evidence from other populations or experimental models. The proposed neuroendocrine and immune-inflammatory pathways—HPA axis dysregulation, chronic low-grade inflammation, and cytokine-mediated effects on mood—are biologically plausible and supported by findings in other chronic pain and inflammatory conditions, as well as by indirect signals in AIS (e.g., pro-inflammatory and pro-oxidative states in scoliotic discs, altered systemic immunity). However, direct evidence measuring cortisol profiles, diurnal rhythms, or inflammatory biomarkers in AIS patients with and without comorbid anxiety or depression is currently lacking, so these mechanisms should be regarded as candidate explanations rather than established pathways. Similarly, the neuropsychological mechanisms of shared pain-emotion circuits derive predominantly from animal models and other chronic pain populations; no direct neuroimaging evidence in adolescents with AIS is yet available. Among interventions, CBT has the strongest direct support in AIS, particularly for brace adherence and perioperative coping, whereas ACT, mindfulness-based stress reduction, and relaxation training remain largely theoretical or extrapolated from other populations, with no AIS-specific trials demonstrating efficacy for anxiety or depression.

A third consideration is that the strength and direction of the evidence vary substantially according to study design, age group, treatment stage, and outcome measured. Cross-sectional studies dominate the prevalence literature, limiting causal inference, while longitudinal data are relatively sparse and often focus on surgical cohorts. Findings in adolescents may not generalize to adults with residual AIS, and vice versa, given differences in body image concerns, pain chronicity, and psychosocial context. Treatment stage matters as well: the psychological profile of patients under observation, during bracing, and in the perioperative period differs meaningfully, and the determinants of distress may shift accordingly. Finally, the choice of outcome—screening positivity versus formal psychiatric diagnosis, generic versus disease-specific QoL instruments, or subjective versus objective biomarkers—substantially influences the magnitude and interpretation of reported associations. These variations underscore the need for standardized, stage-specific, and longitudinally designed research before firm clinical recommendations can be made.

## 11 Limitations

Several methodological limitations of this narrative review should be acknowledged. First, the review is not systematic; a comprehensive search strategy, quality appraisal of individual studies, and meta-analytic synthesis were not performed. As a result, the strength of the conclusions is limited by the selective inclusion of studies, which may not represent the full evidence base. Second, the review’s reliance on English-language publications and on studies from high-income countries may overlook cultural and socioeconomic variations in the experience of AIS and mental health. Third, the review does not quantitatively assess the quality or risk of bias of included studies, so the relative weight given to each finding is based on author judgment. Fourth, no formal framework (e.g., GRADE) was used to grade the certainty of evidence; thus, recommendations regarding screening or intervention should be interpreted with caution. Finally, the broad scope of the topic—spanning psychology, orthopedics, immunology, and health services research—means that some relevant subfields (e.g., neuroimaging, genetic epidemiology) are discussed only briefly, and emerging evidence may have been missed. These limitations underscore the need for a formal systematic review and, ultimately, prospective cohort studies and randomized trials to confirm the associations and mechanisms discussed here.

## 12 Conclusion

The comorbidity of AIS with anxiety and depression represents a significant and multifaceted clinical challenge, characterized by a complex interplay of psychological and physiological factors. This narrative review has highlighted the critical need for a holistic approach to assessment and management, moving beyond the traditional focus on spinal deformity alone. The available literature suggests that body-image disturbance, pain, and social stress are associated with psychological distress in AIS, while neuroendocrine and immune pathways remain plausible candidate mechanisms requiring direct AIS-specific investigation.

From a clinical perspective, while psychosocial assessment is clinically valuable, universal routine screening for all AIS patients cannot yet be mandated in the absence of formal guideline-level evidence. Accordingly, standardized psychosocial screening should be considered as a core component of individualized initial evaluation and longitudinal follow-up. Tools assessing anxiety, depression, body image dissatisfaction, and pain catastrophizing enable early identification of vulnerable patients, allowing for timely intervention before persistent psychological distress impairs treatment adherence and overall clinical outcomes. Balancing orthopedic and psychological care requires clinicians to view patients holistically, recognizing that AIS is a chronic adolescent condition that profoundly impacts self-esteem, social functioning, and long-term QoL.

Effective intervention for AIS-related psychological comorbidities requires a comprehensive, multimodal framework, as no single treatment approach is adequate. Current evidence supports combining targeted psychological interventions, primarily CBT for correcting maladaptive thoughts regarding deformity and pain, with standardized physical management including structured pain control protocols, postural and physiotherapy training, and adjuvant pharmacotherapy when clinically necessary. Mindfulness-based strategies represent a potential adjunctive approach to facilitate emotional regulation; however, dedicated AIS-specific trials are currently lacking, and their clinical value remains speculative, requiring further rigorous evaluation. Crucially, biopsychosocial intervention should integrate family and school environments to reduce external social stressors and establish consistent supportive resources for adolescent adjustment.

Spinal corrective surgery improves spinal biomechanics and may alleviate pain and enhance patient body image, with variable improvements in patient-reported psychosocial outcomes. Nevertheless, individual psychological responses to surgery are heterogeneous, and psychosocial benefits are not inherent to surgical correction alone. Structured preoperative expectation counseling and postoperative psychosocial support can facilitate adaptive recovery and optimize holistic outcomes, whereas unguided recovery may lead to inconsistent psychological outcomes. Surgical teams should therefore incorporate psychological preparedness and mental health recovery into routine perioperative care planning, with recognition that individual patient factors shape final outcomes.

Future research may address existing mechanistic and translational gaps to advance holistic AIS care. Further investigation is needed to clarify the bidirectional pathways linking AIS pathophysiology and affective dysfunction, including potential shared genetic vulnerabilities and the interactions of curve severity, progression patterns, personality traits, and social environments in modulating psychological risk. Such mechanistic insights will support the development of personalized care pathways. Prospective studies are also required to develop and validate feasible, multidisciplinary intervention protocols that improve not only Cobb angle correction but also patient-centered psychological well-being, social participation, and health-related QoL.

In conclusion, contemporary AIS management may benefit from a broader conceptual approach that extends beyond a solely structural corrective model toward an integrated biopsychosocial framework. Anxiety and depression are central, clinically relevant comorbidities in AIS rather than secondary incidental outcomes. Consistent with current evidence, integrating individualized psychosocial assessment, evidence-based physical and psychological interventions, adjunctive supportive strategies under further evaluation, and perioperative holistic care will advance patient-centered AIS management. Further mechanistic research and multidisciplinary protocol validation will continue to refine clinical standards, ultimately improving spinal outcomes and long-term psychosocial resilience among adolescents living with AIS.
